# New comprehensive two-dimensional gas chromatography with sulfur and mass selective detection setup- expanding analytical capabilities for detection of odor active sulfur compounds in food

**DOI:** 10.1007/s00216-026-06449-7

**Published:** 2026-03-25

**Authors:** Lara Skef, David Asen, Sebastiano Pantò, Erich Leitner

**Affiliations:** 1 Graz University of Technology, Institute of Analytical Chemistry and Food Chemistry, Graz, Austria; 2 LECO European Application & Technology Center, Berlin, Germany

**Keywords:** Comprehensive GC × GC, Flow modulation, Sulfur chemiluminescence, Time-of-flight mass spectrometer, Odor active sulfur compounds in food, Solid phase microextraction, Roasted coffee

## Abstract

Volatile sulfur compounds belong to the group of odor active compounds with very low sensory threshold levels and can influence the aroma of various products either in a positive or a negative way. Due to their high odor potency and their low sensory thresholds, it is critical to identify these substances in complex food matrices. A new instrumental setup based on the combination of a two-dimensional comprehensive gas chromatographic system with simultaneous dual detection based on a sulfur chemiluminescence detector and a time-of-flight mass spectrometer separation and identification was tested. Additionally, two different column configurations were tested and compared for further optimization of separation and selectivity. Three different roasted coffee samples were used to demonstrate the potential of this new instrumental configuration, keeping in mind the volatile and odor active fraction of roasted coffee being among one of the most complex volatile composition when comparing to other food and beverage products.

**Graphical Abstract:**

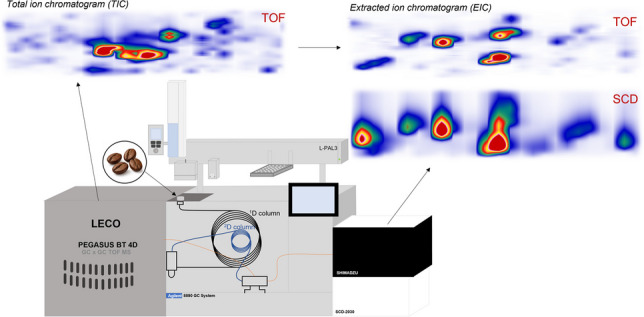

## Introduction

Volatile sulfur compounds (VSCs) constitute only a small portion of total food volatiles, yet they behave as highly potent aroma compounds instilling unique sensory attributes. This diverse class includes thiols, disulfides, polysulfides, thioesters, and sulfur-containing heterocycles, all of which possess unique structural features and specific olfactory properties [1]. Even though they present only a very small fraction of the total volatiles of a product, these compounds can dramatically influence the sensory profile of many foods and beverages [3].

The aroma profile of different foodstuffs represents an important organoleptic attribute that strongly affects consumer experience. During consumption, volatile compounds are being released from food matrix and transported to olfactory epithelium, where they evoke sensory responses. The potency of VSCs mainly arises from their low odor thresholds, often in parts-per-billion (ppb) to parts-per-trillion (ppt) range, most likely reflecting evolutionary pressure to detect these molecules associated with spoilage or degradation [4, 5]. This sensitivity explains why even minor concentrations can enhance the flavor profile, but also lead to formation of off-flavors. In addition to their intrinsic olfactory characteristics, they exhibit synergistic effects arising from interactions with other volatile compounds in food matrices and possible chiral diversity, whereas enantiomers and diastereomers can have lower odor thresholds or display different flavor profiles [6]. Consequently, many VSCs act as character-impact compounds, defining the product identity of specific foods, such as 2-furfurylthiol in coffee, 4-methyl-4-sulfanylpentan-2-one in wine, or diallyl disulfide in garlic [7]. To illustrate the diversity and sensory relevance of these compounds, Table 1 shows some of the VSCs identified in various food matrices, summarizing the wide range of aroma impressions within trace concentrations.

**Table 1:** Commonly identified VSCs in foodstuffs with their respective flavor descriptions and odor thresholds

Name	Formula	Flavor description	Odor threshold water (µg/kg)	Reference
3-Methyl-3-sulfanylbutyl formate	C_6_H_12_O_2_S	Blackcurrant-like, catty, tropical, roasted coffee, sulfury	0.0002–0.0005	[8]
2-Methyl-3-furanthiol	C_5_H_6_OS	Meaty, roasted, savory, chicken-like	0.0004–0.001	[9]
4-Methyl-4-sulfanylpentan-2-one	C_6_H_12_OS	Box tree, passion fruit, broom, black current, catty, rubbery, sulfury	0.0042	[10]
2-Furfurylthiol	C_5_H_6_OS	Roasted, coffee-like, burnt, smoky, meaty, sweet, popcorn-like	0.01	[7, 11]
Ethanethiol	C_2_H_6_S	Onion-like, rubbery, earthy	0.095	[12]
Methanethiol	CH_4_S	Roasted coffee, roasted barley, nuts, rotten cabbage, coffee	0.2	[13]
3-(Methylthio)propanal	C_4_H_8_OS	Boiled potato, baked potato	0.2	[14]
3-Methyl-3-sulfanylbutan-1-ol	C_5_H_12_OS	Broth, onion, sweat, cooked leeks	1.5	[15, 16]
Ethyl 3-(methythio) propanoate	C_6_H_12_O_2_S	Fruity, pineapple	7	[17]
Hydrogen sulfide	H_2_S	Sulfury, rotten eggs	10	[2]

VSCs derive in food from different, complex and often interconnected pathways, mainly shaped by raw materials, processing methods and environmental conditions. Their versatility arises from the fact that a single precursor can yield either identical or entirely different intermediates or products. Conversely, structurally different precursors may generate identical intermediates or products. Intermediates are themselves chemically reactive and may undergo further chemical reactions between themselves or food matrix [18, 19]. Raw material represents the primary source of VSCs, mostly prevalent with sulfur containing amino acids, such as cysteine and methionine. Even though they are precursors in multiple pathways, the chemically most notable are Maillard reactions and Strecker degradation. Cysteine-derived mechanisms are particularly relevant in meat and dairy products, where thermal routes contribute to meaty, roasted or rotten egg aromas [8]. On the other hand, methionine plays a significant role in flavor profile of processed foods, often contributing to creation of off-flavors in processed vegetable, fruit juices, seafood, and fermented beverages (e.g., sake). Alongside thermal reactions, microbial activity significantly enriches the VSC profile in fermented foods such as cheese, wine, beer, and fermented vegetables due to enzymatic degradation of amino acids, peptides, and sulfur-rich secondary metabolites [3].

Among all VSC-rich foods, roasted coffee represents one or the most chemically complex aroma, having identified more than 1,000 volatile compounds. Despite very long list of volatile compounds, literature indicates that only a limited number of compounds are responsible for the aroma of coffee such as pyrazines, furans, aldehydes, ketones, phenols, and sulfur compounds exerting a major effect [20, 21]. The formation of desirable coffee aroma predominantly occurs during the roasting process and continues to further develop during storage. Volatile compounds arise from all coffee constitutes, such as degradation of trigonelline (yielding pyridines), chlorogenic acids (forming vanillin), carotenoids (β-damascenone), lipids (carbonyls). The high temperature during roasting process activates main reactions including Maillard reactions, lipid oxidation and sugar caramelization (forming furaneol) [22]. The complexity of created aroma is influenced by different factors such as species and cultivars, growing and processing parameters and storage conditions [11, 23, 24]. Some of the identified sulfur key aroma compounds in coffee are presented in Table 2.

**Table 2:** Key sulfur-containing aroma compounds identified in roasted coffee and their sensory characteristics

Name	Flavor descriptors	Odor threshold (µg/kg)*
Methanethiol	Sulfur, cabbage-like	0.02–0.5
Dimethyl sulfide	Cooked asparagus-like, putrid	1–3
Dimethyl disulfide	Cabbage-like, sulfuric	1–10
Dimethyl trisulfide	Cabbage-like, sulfuric	0–1-1
2-Fufurylthiol	Roasted coffee, burnt	0.04–0.5
3-Sulfanyl-3-methyl-1-butyl-1-formate	Catty, blackcurrant-like	0.2–2
3-(Methylthio)propionaldehyde	Potato-like, sulfury	1–2

However, during storage secondary reactions, such as oxidation, may lead to additional alterations in the volatile profile, leading to formation of off-flavors or a loss of freshness. VSCs are sensitive to such transformations and therefore they are mentioned as suitable coffee freshness markers [24]. Previous studies have shown that the decline in concentration and stability of thiol compounds is closely related to aroma. Among these compounds, methanethiol is particularly noteworthy being the most abundant thiol in coffee and its presence has been related as an indicator of coffee freshness [24, 25].

In line with their importance, VCSs in food have attracted considerable attention over the past few decades in both food science and industry. A deeper understanding of these compounds would significantly contribute product quality, optimization, and processing strategies of these products. However, due to extremely low odor thresholds, minute concentrations, high chemical reactivity, thermal instability, and tendency to co-elute with other volatiles their analytical determination remains highly challenging [4]. Furthermore, the structural diversity of VSCs requires powerful, highly sensitive and selective analytical systems and methods for their identification. Gas chromatography (GC) has long been used as the primary technique for VSC determination because of its superior resolution and compatibility with complex food matrices. To enhance selectivity towards sulfur-containing compounds it would be further coupled with sulfur-selective detectors such as the flame photometric detector (FPD), pulsed flame photometric detector (pulsed FPD), and the sulfur chemiluminescence detector (SCD). Even though conventional one-dimensional GC is routinely used in analysis of volatile fraction in food and beverages, it has a drawback of limited peak capacities and persistent co-elution issues. Achieving maximal separation often requires long run times yes still cannot fully resolve overlapping peaks, particularly since volatiles are always present in trace amounts [11]. Consequently, VSCs are often underestimated or even overlooked in such analyses. Literature search revealed several studies combining GC with mass spectrometry (MS) for analysis in various coffee matrices, including green and roasted beans, ground coffee powder, brewed coffee, and espresso [26–31]. However, the summarized findings indicate that only a limited number of sulfur compounds were identified in these studies. Further methodological refinements, including coupling MS with flame ionization detector (FID) and olfactometry (O), increased the number of identified sulfur species to approximately 20–25 compounds in selected investigations [32–34].

By contrast, comprehensive two-dimensional gas chromatography (GC × GC) offers two orthogonal separations and increased peak capacity. This significantly improves resolution of complex mixtures and enables the simultaneous use of complementary detection systems within a single analytical run. The enhanced separation efficiency of GC × GC also helps in overcoming co-elution issues and improves sensitivity and selectivity. Table 3 summarizes selected GC × GC- and multidimensional gas chromatography (MDGC)-based methods reported in the literature used for analysis of coffee matrices, including detector configurations, extraction techniques, and key analytical findings. Most studies focused on volatile profiling, identification of large number of compounds, and investigation of roasting effects, origin differences, and aroma formation pathways for better understanding of aroma complexity. Quantitative method validation was so far not a central objective, and limit of detection (LOD) and limit of quantification (LOQ) values were not determined, with only one study reporting such data for two selected sulfur compounds [36]. The reason for that is mainly based on the extreme chemical complexity of coffee, where analytical efforts have largely prioritized maximizing separation capacity and compound identification over rigorous quantitative validation of individual sulfur species.

**Table 3:** Literature review of GC × GC and MDGC based methods for identification of VOCs in coffee

GC separation	Detector	Sample preparation	Matrix	Key findings	Reference
GC × GC MDGC	FID, O, MS	SPME	Ground coffee	11 odor volatile compounds out of which 2 sulfur containing compounds	[35]
GC × GC; GC	TOFMS-MS; O	SPME	Roast and ground coffee, filter coffee brew	3-Methyl-2-butene-1-thiol, 3-(methylthio)propionaldehyde (methional)	[36]
GC, GC × GC	MSM; TOFMS	SPME	Barley coffee (coffee substitute)	With GCxGC setup 64 volatiles have been identified; none of which sulfur	[37]
GC × GC	TOFMS	HS-SPME	Coffee powder, espresso coffee brew	390 volatiles identified including 17 chemical families, out of which 12 sulfur compounds	[38]
GC × GC	TOFMS: qMS	SPME	Coffee beans	> 1000 peaks processed, 44 selected for semi-quantitative analysis (1 sulfur compound)	[39]
GC × GC	FID, TOFMS	CSC and XAD-2 sorbents	Coffee beans	> 1000 peaks detected; 22 compounds identified; no sulfur compounds reported	[40]
GC GC × GC	TOFMS	HS-SPME	Coffee beans, powder and brew	GC: ~ 100 compounds (3–5 sulfur); GC × GC: 175–374 compounds (10–25 sulfur)	[41]
GC × GC	TOFMS	HS-SPME	Roasted coffee powder	378 volatiles identified, 27 sulfur compounds	[42]
GC × GC	TOFMS	DHS	Roasted coffee beans	169 volatiles identified; few sulfur compounds (not specified)	[43]

Despite these advances, only a limited number of studies have applied GC × GC to sulfur analysis in coffee, and to the best of our knowledge, none have reported the combination of GC × GC with simultaneous TOFMS and SCD detection. To address this analytical gap, in this work, a new instrument based on a flow-modulated comprehensive two-dimensional gas chromatography system equipped with a fixed splitter and dual detection is presented. By combining the universal TOF and a SCD facilitates simultaneous profiling and sulfur compounds detection in a single run. This integrated approach shows improved identification and confirmation of sulfur compounds in complex food matrices, as demonstrated here for roasted coffee.

## Materials and methods

### Instrument set up

**Fig. 1:**
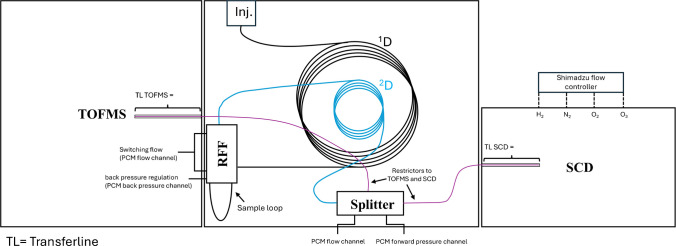
Schematic diagram of the new GC × GC-TOFMS/SCD setup

GC × GC analyses were performed on an 8890 gas chromatograph from Agilent Technologies (Santa Clara, CA) equipped with a reverse fill/flush (RFF) flow modulator [44] and splitter from LECO (Paradigm & Shift™, LECO Corporation, St. Joseph, MI, USA), adapted in this work to allow flow splitting between a TOFMS, operating under vacuum (Pegasus BT-4D, LECO Corporation, St. Joseph (MI), USA) and an SCD operating at 1.2 kPa (Nexis SCD-2030, Shimadzu, Kyoto, Japan). An L-PAL 3 autosampler from LECO with automated solid phase microextraction (SPME) capabilities was used for automated injections. Worth of notice, the SCD detector gasses and flows (i.e., H_2_, N_2_, O_2_ and, O_3_) were controlled via the Shimadzu hardware and software. Two different column setups were investigated. For both setups, a Rxi-5MS (Restek) 19.95 m × 180 µm × 0.18 µm was employed as ^1^D column. Both the selected ^2^D columns had the same dimensions, i.e., 3.65 m × 250 µm × 0.25 µm and mid-polar crossbond stationary phases. However, column selection 1 utilized a Rxi-17Sil MS (Restek, Bellefonte, USA), and column selection 2 an SH-200 MS (Shimadzu, Kyoto, Japan). The RFF sample loop was an uncoated 0.17 m × 530 µm metal capillary. The detector splitter used a total length of 0.80 m × 125 µm (0.49 m in the main oven and 0.31 m in the TOFMS transferline) and 0.59 m × 320 µm (0.34 m in the main oven + 0.25 m in the SCD transferline) deactivated capillaries from Trajan Scientific and Medical (Victoria, AUS). For method setup and data acquisition LECO ChromaTOF (Version 5.57.23) was used. Constant flow rate was attained through linear pressure ramps calculated by ChromaTOF software and based on standard Hagen-Poiseuille flow equation, which considers temperature, pressure, and capillary dimension. A proprietary flow correction procedure has been performed after the installation of each column selection to adjust the auxiliary flow of the pressure control module (PCM) to account for variations in dimensions of the columns, which impact the flow calculations. The correction was necessary to ensure proper modulation avoiding over filling and/or under flushing during modulation but also to maintain a constant split ratio between TOFMS and SCD detectors. A schematic diagram of the GC × GC-TOFMS/SCD is displayed in Fig. 1.

### Sample preparation

Three types of ground roasted coffee samples with different best before dates and packaging regimes were selected for first evaluation of the potential new system setup.

The first sample were whole roasted beans from a small-scale local producer packed in an alumina coated plastic bag with an aroma protection valve and a given shelf life of May 2025. The beans were ground with a laboratory tube mill at 5000 rpm for 10 s (IKA, Staufen, Germany) immediately before the measurements. Coffee sample two was a 250 g vacuum sealed, ground coffee in an alumina coated plastic bag bought in a local supermarket. The shelf life was labeled to be May 2026. The third sample, also bought in the local supermarket, was a ground espresso packed in an alumina coated plastic bag with an aroma protection valve and a shelf life until April 2026.

For each sample 100 mg of ground coffee was weighed in 20-mL headspace vials and closed with magnetic crimp cap with PTFE lined silicone septum. Each sample was prepared in triplicates.

### Sample enrichment and GC × GC analysis

The enrichment of the samples was executed in the L-PAL 3 autosampler agitator with a speed of 250 rpm. Samples were first preheated for 5 min at 40 °C. Volatile compounds were then extracted for 5 min at 40 °C using a 1 cm PAL 3 smart fiber with a 50/30 µm Divinylbenzene/Polydimethylsiloxane/Carbon wide range coating (CTC, Zwingen, Switzerland), followed by injection into the injector port at 260 °C in split mode with a split ratio of 5:1.

The same temperature program was used for all measurements. The initial oven temperature was held at 40 °C for 1 min, ramped at 5 °C/min to 190 °C, where it was held for 1 min. The total run time was 32 min. The modulation period P_m_ was set to 5 s, with a re-injection time of 200 ms, automatically calculated by the software to provide a flush volume of 1.76 times the fill volume to ensure full transfer conditions and account for the diffusion of the analytes through the sample loop. Helium (99.999%) was used as a carrier gas at a flow rate of 0.5 mL/min for ^1^D and 30 mL/min for ^2^D. The TOFMS transfer line and ion source were heated to 345 °C and 300 °C, respectively. Electron ionization was selected at 70 eV, with an acquisition rate of 100 Hz from 30–500 amu. The SCD interface temperature was set to 200 °C, with the furnace temperature set to 850 °C. Gas flow rates for the SCD were set as following: 80 mL/min for H_2_, 40 mL/min for N_2_, 10 mL/min for O_2_, and 25 mL/min for O_3_. Sampling rate was set to 125 Hz. The flow splitter delivered 1.03 mL/min to TOFMS with the remainder of the flow (~ 73.1 mL/min) to the SCD.

### Data analysis

Same as for method setup LECO ChromaTOF (Version 5.57.23) was used for data processing and data export of TOFMS data, whilst Shimadzu LabSolution Version 5.132 was used for the SCD data. All measured data were exported in.cdf file format and imported into ChromSpace + (SepSolve, Offenbach am Main, Germany) version 2.2 for direct alignment of the TOFMS and SCD data sets. The same software was also used in preparation of all figures presented in this study. Peak identification for the TOFMS trace was performed with LECO ChromaTOF, using the NonTarget Deconvolution® peak find algorithm. Positive matches were assigned to any peak with a minimum signal-to-noise (S/N) ratio of 20, minimum stick count of 6, and a minimum similarity score of 650 when compared to the NIST library 2023. Linear retention indices (LRIs) were calculated in the range of 700–1400 using n-alkanes identified in one of the analyzed samples. The calculated LRIs were compared with values reported in the literature and evaluated for consistency with increasing retention time.

## Results and discussion

To comprehensively evaluate the system performance, two different column configurations were compared with regard to their ability to achieve separation in the second dimension of the GC × GC system. For each column set, triplicates were measured to ensure reproducibility and robustness. The resulting datasets were processed under identical conditions to allow direct comparison of chromatographic performance.

The volatile compounds obtained from each column configuration were evaluated, using several parameters of interest as performance indicators: *total peak number*, the number of tentatively *identified compounds* (noting it may contain false positive, and/or duplicate identifications), and number of *identified sulfur compounds, defined as compounds whose assigned names contained* “thi” (e.g., *thi*ols, *thi*ophenes, *thi*azoles, …), “sulf”, or “mercap”. The summarized results are presented in Table 4.

**Table 4:** Comparison of the two column selections in produced peaks on the TOFMS trace

Column selection	^2^D column type	Total peaks	Identified compounds	Sulfur compounds
1	Rxi-17Sil MS	811 ± 43	499 ± 21	32 ± 6
2	SH-200 MS	1223 ± 41	808 ± 17	68 ± 3

Both column configurations provided extensive peak separation in the two-dimensional space, demonstrating the general suitability of the GC × GC-TOFMS/SCD setup for the analysis of complex coffee volatiles. However, column selection 2, employing the SH-200MS column in the second dimension, showed enhanced separation performance in the TOFMS trace. This improvement was reflected in a higher total peak count, an increased number of tentatively identified compounds and a greater number of identified sulfur-containing species. The improved separation can be attributed to the higher polarity of the SH-200MS stationary phase, which enhanced the distribution of analytes across the second dimension.

In contrast, SCD trace showed minimal variation in total peak number between the two column selections. This observation is consistent with the element-selective detection principle of the SCD, which responds exclusively to sulfur-containing species. Because the SCD does not detect the majority of other non-sulfur volatiles present in coffee, overall peak density remains low, and chromatographic congestion is reduced. Consequently, changes in second-dimension has less influence on total peak count in the SCD trace.

Based on the superior separation efficiency and therefore improved identification performance observed in the TOFMS data, column configuration 2 was selected for further data interpretation.

**Fig. 2:**
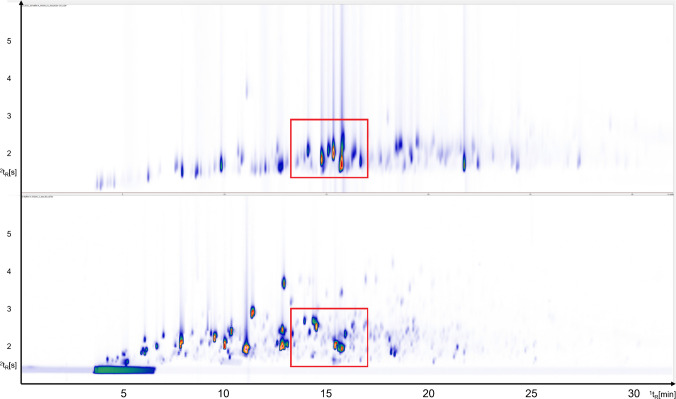
Complete GC × GC chromatogram of freshly roasted coffee with SCD (top) and TOFMS (bottom). The region highlighted in red indicates enlarged area in Fig. 3

The comparison of SCD and TOFMS trace presented in Fig. 2 highlights both the chemical complexity of sulfur-containing compounds, and the abundance of other non-sulfur volatile compounds in roasted coffee. The TOFMS trace (Fig. 2, bottom) reflects the full compositional diversity of the coffee matrix, including furans, pyrazines, aldehydes, ketones, phenols, esters, and sulfur compounds. In contrast, the SCD trace (Fig. 2, top) selectively visualizes sulfur-containing compounds, thereby drastically reducing chromatographic complexity and allowing focused evaluation of VSCs.

**Fig. 3:**
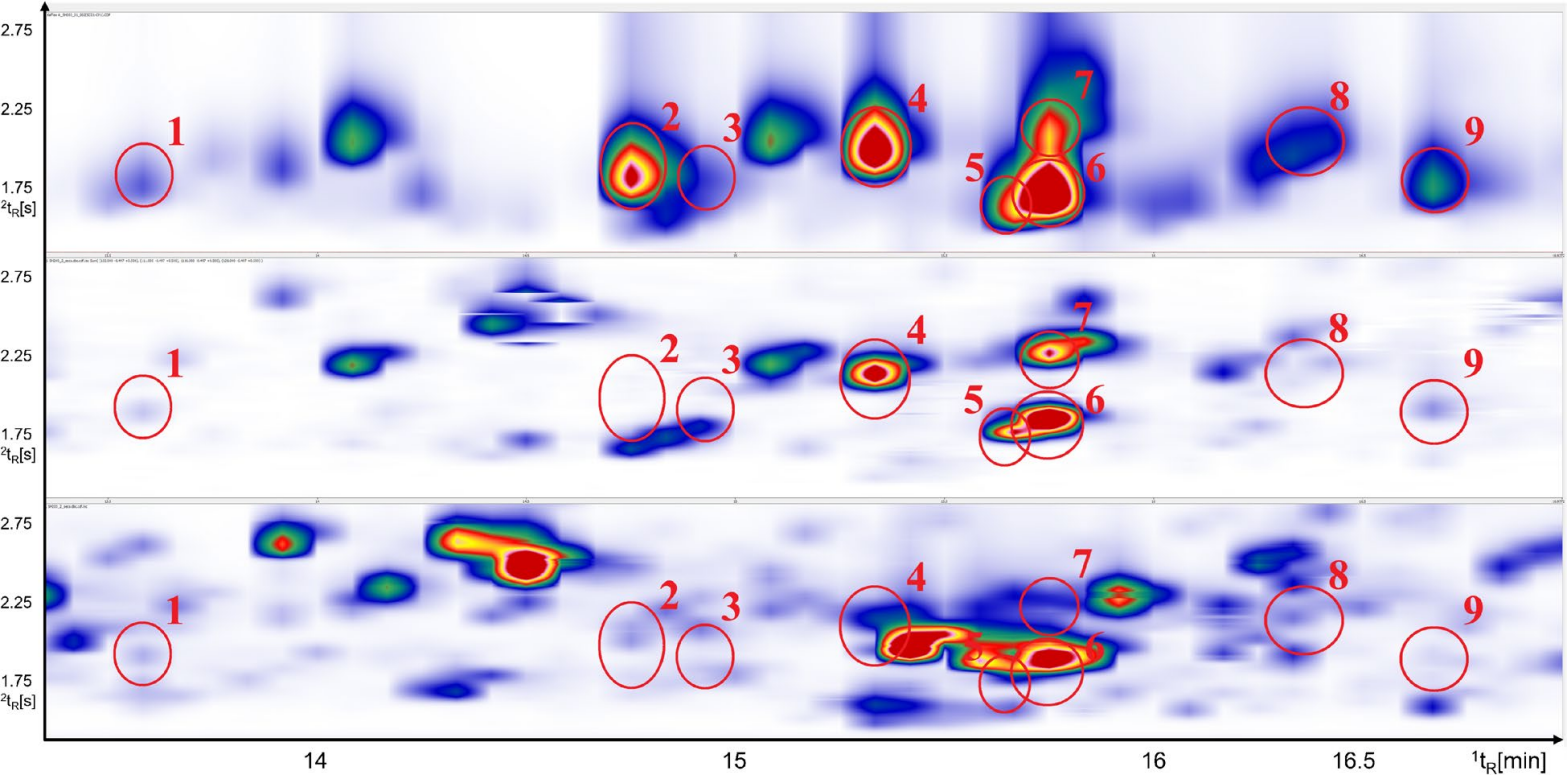
Detailed view of the part of chromatogram for freshly roasted coffee (^1^t_R_ 13.3–17.0 min; ^2^t_R_ 1.4–2.9 s); top to bottom: SCD, EIC (m/z 102, 111,116, 128 each ± 0.5 Da), and TIC

As expected, SCD shows higher signal intensities observed for low-concentration sulfur compounds compared to the corresponding signals in the TOFMS trace. This difference becomes especially evident in the highlighted zoomed region in Fig. 3 where the SCD trace (top) is compared to the extracted ion chromatogram (EIC) and total ion chromatograph (TIC). In the TIC, sulfur compounds appear only as small peaks or are partly hidden by signals from more abundant compounds (e.g., pyrazines). In contrast, the SCD chromatogram shows these sulfur compounds clearly and with much higher relative intensity. This demonstrates the higher sensitivity and selectivity of the SCD for detecting trace-level sulfur species. Further on, the increased separation efficiency achieved in the ^2^D significantly enhances better deconvolution of otherwise co-eluting peaks. That can be clearly seen in Fig. 3 (bottom). Peaks numbered 5–7 co-elute with C_3_-substituted pyrazines, which are present in such high concentrations that they dominate and completely mask the sulfur compounds of interest in the TOFMS trace proving confident identification based just on the TIC challenging. However, the corresponding SCD trace (Fig. 3, top), reveals the presence of VSCs withing the same retention region by eliminating aforementioned interferences from non-sulfur species. This comparison demonstrates the analytical strength of using SCD and TOFMS parallelly. While TOFMS provides structural information and broad profiling capability, SCD confirms the presence of sulfur-containing compounds, retention times and identification correctness confirmation with high sensitivity and selectivity.

To verify that, selective EICs were generated from TOFMS trace. Mass fragments characteristic of sulfur-containing species but not present in pyrazines were selected (m/z 102, 111,116, 128 ± 0.5 Da). Extraction of these ions (Fig. 3, middle) revealed presence of at least three distinct sulfur-containing compounds. For compounds numbered 1, 3, 8, and 9 no selective m/z fragments were extracted in order to avoid introducing of potential interferences.

The sulfur compounds numbered in Fig. 3 were also identified in the SCD trace, providing orthogonal validation of their presence. A summarized list of these identified sulfur compounds is presented in Table 5.

**Table 5:** Numbered sulfur compounds identified in Fig. 3, with respective CAS number, formula, flavor descriptors, literature LRI and calculated LRI on semi-standard non-polar column

	**Name**	**CAS**	**Formula**	**Flavor descriptors**	**Library LRI**	**Calculated LRI**
1	4,5-Dimethylthiazole	3581–91-7	C_5_H_7_NS	Roasted, nutty, green; cereal, toasted, musty, mushroom, shellfish	935 ± 7	938
2	3-Methyl-3-sulfanyl-1-butanol	34300–94-2	C_5_H_12_OS	Cat pee	970 ± 0	974
3	2-Methoxy-5-methylthiophene	31053–55-1	C_6_H_8_OS	n.d	n.d	980
4	Dihydro-2-methyl-3(2H)-thiophenone	13679–85-1	C_5_H_8_OS	Green burnt coffee note	996 ± 6	992
5	2,4,5-trimethylthiazole	13623–11-5	C_6_H_9_NS	Chocolate, nutty, coffee aroma	997 ± 2	1000
6	2-[(Methylthio)methyl]- furan	1438–91-1	C_6_H_8_OS	Coffee like and strong, mustard, garlic, burnt flavor	998 ± 3	1002
7	2-Thiophenecarbaldehyde	98–03-3	C_5_H_4_OS	Benzaldehyde like note	1008 ± 7	1005
8	2-Acetylthiazole	24295–03-2	C_5_H_5_NOS	Nutty, hazelnut, popcorn, roasted peanuts	1021 ± 5	1023
9	2-Thiophenemethanol	636–72-6	C_5_H_6_OS	Savory, roasted coffee, coffee	1043 ± 13	1034

For the region of very volatile low boiling compounds, the three coffee samples clearly show differences in relative concentrations in the SCD trace. It is particularly pronounced for methanethiol widely recognized as a freshness indicator, which appears as the first detectable sulfur compound in the SCD trace [25]. Also, the benefit of selective nature of sulfur selective detection is clearly visible in this early retention time region when compared to the TOFMS trace. In the first minutes of the chromatogram this region is dominated by massive interference, mainly deriving from air introduced during the injection process which superimposes the signals from the early eluting compounds.

**Fig. 4:**
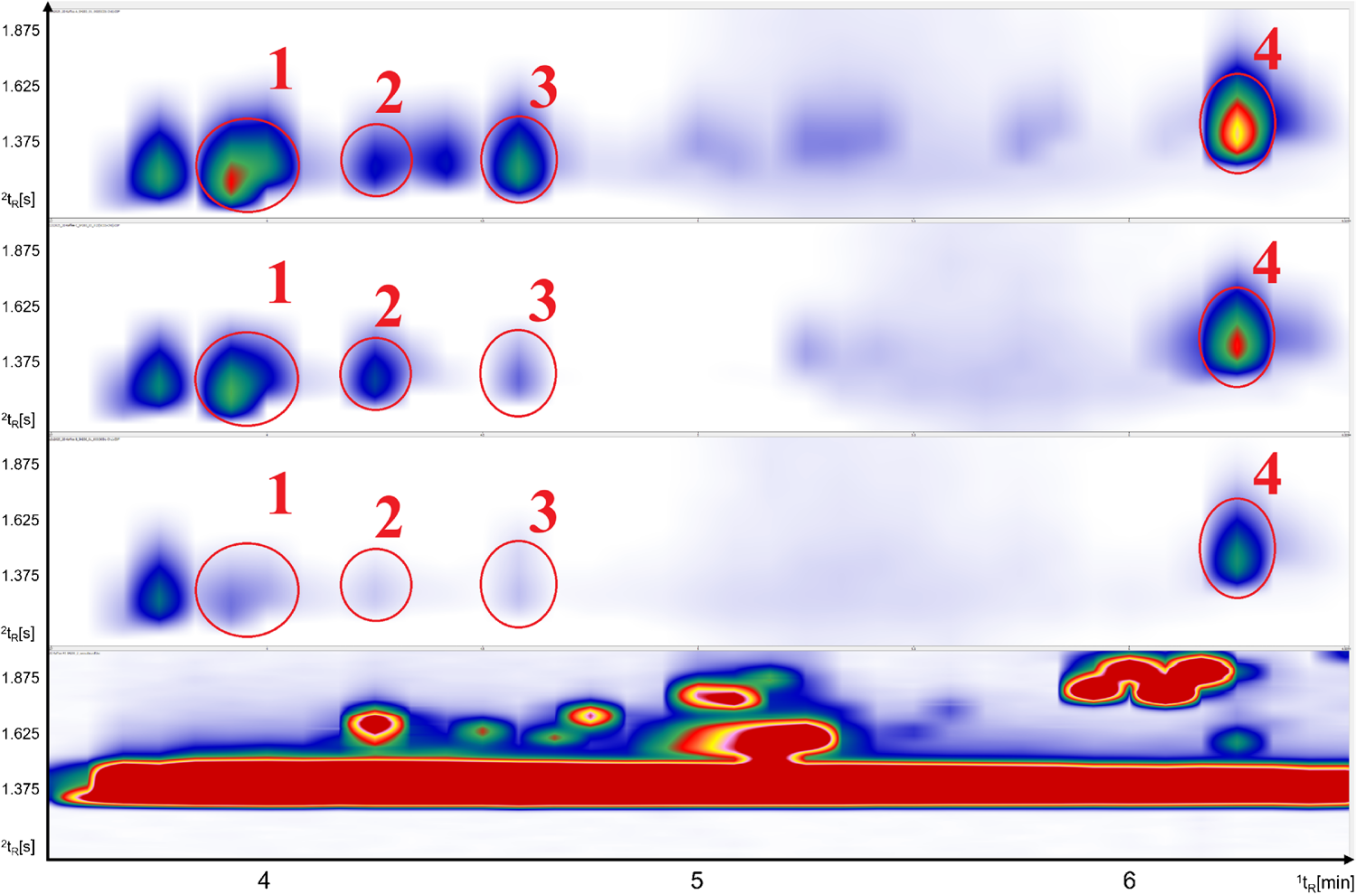
^1^t_R_ 3.5–6.5 min; ^2^t_R_ 1.0–2.0 s; top to bottom SCD trace: freshly roasted coffee, ground espresso in aroma protection packaging, vacuum-packed ground coffee; bottom: TOFMS trace of freshly roasted coffee. 1: methanethiol; 2: dimethylsulfide; 3: carbon disulfide; 4: thiophene

This clear difference in the early-eluting region also reflects the sensitivity characteristics of the system (Fig. 4). From a sensitivity perspective, it should be considered that the performance observed here results from the combined influence of instrumental configuration and sample preparation. Coffee was intentionally selected because of its chemical complexity and the challenging nature of its volatile fraction, providing a demanding test matrix for evaluating system performance. Headspace SPME was applied, and its performance depends on several parameters, including fiber type, extraction temperature, extraction time, matrix composition, and analyte partition behavior. Consequently, the analytical sensitivity cannot be attributed solely to the instrumental setup but represents the combined effect of extraction and detection. For the same reason, recovery values were not determined, as systematic optimization and validation of extraction parameters were beyond the scope of this work.

To evaluate detector sensitivity independently of extraction parameters, additional experiments were performed using liquid injection of sulfur standards. Liquid injection allows defined amounts of analytes to be introduced directly into the system, eliminating variability related to headspace partitioning and fiber adsorption. Under these controlled conditions, the system achieved a LOD of 1.45 pg of sulfur/s, demonstrating the high sensitivity of the presented GC × GC-TOFMS/SCD configuration.

## Conclusions

Volatile sulfur compounds represent a small portion of odor active compounds yet possessing powerful impact due to their very low sensory threshold values. An additional property as minute concentrations, high chemical reactivity, thermal instability, and co-elution tendency makes them particularly challenging for analytical determination. Conventional analytical approaches typically use one-dimensional GC coupled to one single detector. While suitable for general volatile profiling, using such systems often leads to limited separation capacity and insufficient selectivity, especially in resolving sulfur species in complex food matrices. Co-elution with high-abundance compounds and matrix interferences frequently leads to underestimation or misidentification of VSCs. In this study, we evaluated a newly developed instrumental setup based on a flow-modulated GC × GC system equipped with simultaneous dual detection using TOFMS and SCD. Measurement performed on two coffee samples by using the combined approach clearly demonstrated superior profiling of VSCs. Our results show enhanced chromatographic separation performance due to increased peak capacity, improved sensitivity for VSCs via SCD, and greater identification of compounds compared to conventional one-dimensional detection. The integration of SCD allowed detection of sulfur species present at trace-level concentrations even in regions dominated by high-abundance non-sulfur volatile compounds, while parallel TOFMS acquisition enabled structural interpretation and library-based identification.

Furthermore, the introduction of cryogenic oven cooling could significantly enhance the retention and detectability of very volatile sulfur species as methanethiol, which are frequently underestimated or entirely overlooked by using conventional temperature programs.

As aforementioned, this study represents an initial evaluation of the new system using a highly complex food matrix. It should be emphasized that the primary objective was not to perform full method validation, but to assess and demonstrate the capability of the newly implemented GC × GC-TOFMS/SCD setup to maximize separation efficiency and expand compound identification in a challenging matrix. Coffee was intentionally selected due to its complex volatile composition, making it a suitable for assessing the performance of the system in detecting and resolving trace-level sulfur compounds. This means additional optimization of method parameters including column selection, temperature program, modulation conditions remain necessary to achieve full chromatographic potential and robust performance of the system to ensure reproducibility. In addition, detailed characterization of the splitter configuration coupled to the SCD with a series of sulfur standards should be performed to ensure a constant split ratio over the applied temperature range of the method.

Nevertheless, the presented work represents a substantial advancement for further development of analytical methodologies in volatile sulfur profile characterization. The demonstrated combination of comprehensive two-dimensional separation with parallel universal and sulfur-selective detection provides a powerful platform for investigation of sulfur-driven aroma chemistry in coffee and other complex food matrices. This approach opens new opportunities for improved understanding of freshness, quality differentiation, and process-induced sulfur chemistry.

## Data Availability

The data supporting the findings of this study are included in the article. Additional data are available from the corresponding author upon reasonable request.
